# WW Domain Containing E3 Ubiquitin Protein Ligase 1 (WWP1) Negatively Regulates TLR4-Mediated TNF-α and IL-6 Production by Proteasomal Degradation of TNF Receptor Associated Factor 6 (TRAF6)

**DOI:** 10.1371/journal.pone.0067633

**Published:** 2013-06-17

**Authors:** Xiao-Wen Lin, Wei-Cheng Xu, Jian-Gang Luo, Xue-Jiao Guo, Tao Sun, Xu-Li Zhao, Zhi-Jian Fu

**Affiliations:** 1 Department of Pain Management, Provincial Hospital affiliated to Shandong University, Shandong University, Jinan, P.R. China; 2 Department of Pain Management, Provincial Hospital affiliated to Shandong University, Jinan, China; 3 Department of Orthopedics, Provincial Hospital affiliated to Shandong University, Jinan, China; Aristotle University of Thessaloniki, Greece

## Abstract

**Background:**

Toll-like receptors (TLRs) play a pivotal role in the defense against invading pathogens by detecting pathogen-associated molecular patterns (PAMPs). TLR4 recognizes lipopolysaccharides (LPS) in the cell walls of Gram-negative bacteria, resulting in the induction and secretion of proinflammatory cytokines such as TNF-α and IL-6. The WW domain containing E3 ubiquitin protein ligase 1 (WWP1) regulates a variety of cellular biological processes. Here, we investigated whether WWP1 acts as an E3 ubiquitin ligase in TLR-mediated inflammation.

**Methodology/Results:**

Knocking down WWP1 enhanced the TNF-α and IL-6 production induced by LPS, and over-expression of WWP1 inhibited the TNF-α and IL-6 production induced by LPS, but not by TNF-α. WWP1 also inhibited the IκB-α, NF-κB, and MAPK activation stimulated by LPS. Additionally, WWP1 could degrade TRAF6, but not IRAK1, in the proteasome pathway, and knocking down WWP1 reduced the LPS-induced K48-linked, but not K63-linked, polyubiquitination of endogenous TRAF6.

**Conclusions/Significance:**

We identified WWP1 as an important negative regulator of TLR4-mediated TNF-α and IL-6 production. We also showed that WWP1 functions as an E3 ligase when cells are stimulated with LPS by binding to TRAF6 and promoting K48-linked polyubiquitination. This results in the proteasomal degradation of TRAF6.

## Introduction

Toll-like receptors (TLRs) play a pivotal role in defense against invading pathogens through their detection of pathogen-associated molecular patterns (PAMPs) [1]–[3]. Inappropriate activation or over–activation of TLR signaling is believed to contribute to inflammatory disorders such as septic shock and autoimmune diseases [4]. Thus, it is critical to identify negative regulators of TLR signaling and determine the mechanisms by which TLR signaling is controlled.

TLR4, the best characterized member of this family, recognizes lipopolysaccharides (LPS) in the cell walls of Gram-negative bacteria [5], [6]. TLR4 signaling consists of two distinct pathways: the myeloid differentiation factor 88 (MyD88)-dependent pathway and the TIR domain-containing adaptor-inducing IFN-β (TRIF)-dependent pathway [7].The recognition of LPS by TLR4 recruits adaptors, including MyD88, IL-1 receptor-associated kinases (IRAK), and TNF receptor-associated factor 6 (TRAF6) [8], to the infection site which then triggers the activation of a number of intracellular signaling cascades. These signaling cascades include the NF-κB and mitogen-activated protein (MAP) kinase (ERK, JNK and P38) pathways [9]. The activation of these signaling pathways, in turn, alters the biosynthesis of immunoregulatory molecules such as tumor-necrosis factor-α (TNF-α) and interleukin 6 (IL-6) [10]. Additionally, the recruitment of TRIF promotes the activation of the TRAF3/NAP1 pathway and, subsequently, activation of TBK1/IκB kinase ε (IKKε) which results in the phosphorylation, dimerization, and nuclear translocation of IRF3 as well as the induction of IFN-β [11], [12].

The WW domain containing E3 ubiquitin protein ligase 1 (WWP1) belongs to the C2-WW-HECT type E3 family which is comprised of eight other members, including NEDD4, AIP4/Itch, SMURF1, and SMURF2 [13]–[16]. WWP1 contains an N-terminal C2 domain, four tandem WW domains for substrate binding, and a C-terminal catalytic HECT domain for ubiquitin transfer [17]. WWP1 interacts with a variety of substrate proteins, including Smad2 [18], ErbB4/HER4 [19], RNF11 [20], SPG20 [21], KLF2 [22], p63 [23], and RUNX2 [24]. As a ubiquitin E3 ligase, WWP1 regulates the expression levels and activities of these and other substrate molecules. Consequently, WWP1 regulates many cellular biological processes, including protein trafficking and degradation, signaling, transcription, and viral budding. WWP1 has also been implicated in signaling involved in cancer, neurological diseases, infectious disease responses, and aging [25]. A recent report by Yang et al. (2013) suggests that WWP2 mediates K48-linked ubiquitination and degradation of TRIF upon TLR3 activation, thus acting in the TLR3-mediated innate immune response [26]. Although WWP1 is similar in structure and function to WWP2, the role of WWP1 in TLR-mediated inflammation has not been well studied. Thus, in this study, we investigated the role of WWP1 as an E3 ubiquitin ligase in TLR-mediated inflammation.

In the present study, we showed that WWP1 negatively regulates the TNF-α and IL-6 production stimulated by LPS. These proteins are produced downstream of the interaction between WWP1 and TRAF6 which enhances the K48-linked polyubiquitination of TRAF6, resulting in its proteasomal degradation.

## Materials and Methods

### Cell culture and reagents

RAW264.7 cells and HEK293T cells were purchased from ATCC (ATCC® TIB-71™, ATCC® CRL-1573™), and maintained in Dulbecco's modified Eagle's medium (DMEM; Hyclone) supplemented with 10% (v/v) heat-inactivated FBS (Gibco) and 100 U/mL penicillin and streptomycin. The peritoneal macrophages were cultured in RPMI-1640 medium supplemented with 10% (v/v) FBS and 100 U/mL of penicillin and streptomycin.

The following antibodies were used: rabbit anti-phospho-IRF-3 (Ser396) (4947), rabbit anti-phospho-IκBα (Ser32/36) (9246), rabbit anti-IRF-3 (4302), rabbit anti-phospho-NF-κB p65 (Ser536)(3033), rabbit anti-NF-κB p65 (4764), rabbit anti-p38 (9212), rabbit anti-phospho-Erk1/2 (9101), rabbit anti-phosphorylated p38 (9215), and rabbit anti-phospho-Jnk (9251) (Cell Signaling Technology); rabbit ant-WWP1 (sc-100679), rabbit anti-IgG (sc-66931), mouse anti-TRAF6 (sc-8409), mouse anti-IRAK1 (sc-55530), rabbit anti-TLR4 (H-80) (sc-10741), protein G-agarose (sc-2002), and HRP-conjugated secondary Abs (Santa Cruz Biotechnology); mouse anti-GAPDH (TA-08), mouse anti-His (TA-02), and mouse anti-Myc (TA-01) (ZSGB-BIO); rabbit anti-Ub (AB1586), rabbit anti-Lys63 specific Ub (05-1038), and rabbit anti-Lys48 specific Ub (AB1307) (Merck Millipore). MG132 (C2211) and LPS (*Escherichia coli*, L4005) were purchased from Sigma and mouse TNF-α (410-MT-010) from R&D Systems.

### Plasmid construction, RNA-mediated interference, and transfection

The plasmids expressing Myc-TLR4 (RC206488), TRIF (SC107526), and MyD88 (SC327786) were manufactured by OriGene and were kindly provided by Prof. Hu Xia at QiLu Hospital, Shandong University. The cDNAs encoding TRAF6 and IRAK1 were inserted into the pCMV-N-Myc vector, and cDNAs encoding WWP1and WWP1-C890A mutant (C890A) were inserted into the pcDNA3.1-Hisc vector. The following primers were used: WWP1: sense 5′-ATGGCCACTGCTTCACCAAGATCTG-3′ and anti-sense 5′-TCATTCTTGTCCAAA TCCCTCTGTC-3′; C890A: sense 5′-AGCTTTTAATCGCT-3′ and anti-sense 5′-GTATGGCTTCTTGG-3′; TRAF6 sense 5′-ATGAGTCTCTTAAACTGTGAGAACA-3′ and anti-sense 5′-CTACACCCCCGCATCAGTACTTCGT-3′; and IRAK1 sense 5′-ATGGCCGGGGGGCCGGGCCCCGGGG-3′ and anti-sense 5′-TCAGCTCTGGA ATTCATCACTTTCT-3′.

The siRNAs targeting WWP1 messenger RNA (mRNA) and a scrambled siRNA used as a negative control (NC) were designed using the Ambion website. These siRNA were delivered using lentivirus particles (1 ml, 10^8^ TU/ul) by GenePharma (Shanghai GenePharma Co., Ltd., Shanghai, China). The WWP1 siRNA (LeshWWP1) sequence was 5′-GAGTTGATGATCGTAGAAG-3′. The NC (LeshNC) sequence was 5-TTCTCCGAACGTG TCACGT-3.

Transfected RAW264.7 were stably selected using G418 (600 ng/mL) and pooled for further experiments. For transient silencing, lentiviral siRNA was injected into the medium of cultured macrophages.

### Immunoprecipitation

Cells were scraped from the plate and lysed with 300 µL of radio immune precipitation assay buffer containing a protease inhibitor mixture for 30 min on ice. Lysates were pre-cleared by incubation with 20 µL of 50% G-agarose (sc-2002) for 10 min. The lysates were then centrifuged at 3,000×g for 5 min at 4°C. The supernatant and antibody were gently rocked overnight at 4°C. A 50% slurry of G-agarose (sc-2002) (30 µL) was then added and incubated for 5 h at 4°C. Precipitates were washed four times with 800 µL of radioimmune precipitation assay buffer and then resuspended in 50 µL of 2×SDS loading buffer. The samples were boiled for 5 min and then analyzed by Western blotting.

### Mouse

C57BL/6J mice were obtained from Joint Ventures Sipper BK Experimental Animals (Shanghai, China). All animal experiments were undertaken in accordance with the National Institutes of Health Guide for the Care and Use of Laboratory Animals and with the approval of the Scientific Investigation Board of the Medical School of Shandong University (Jinan, Shandong Province, China).

### Isolation of Macrophages

Peritoneal macrophages were harvested from 6-week old mice 4 days after thioglycollate (BD, Sparks, MD) injection. Ice-cold PBS supplemented with 20 U/mL heparin and 1 mM EDTA was used to wash the peritoneal cavity by lavage. Peritoneal macrophages were isolated and cultured in endotoxin-free DMEM medium with 10% (vol/vol) FBS (Invitrogen) and 100 U/mL penicillin and streptomycin for 24 h in 5% CO_2_ at 37°C. Non-adherent cells were removed by washing three times with ice-cold PBS and adherent cells were counted for use in experiments.

### Immunoblot Analysis

Cells were washed with PBS, collected, and lysed for 30 min on ice using a modified radioimmune precipitation assay buffer (Applygen Technologies Inc., Beijing, China) containing a protease inhibitor mixture (Fermentas). Cell lysates were sonicated and then centrifuged at 15,000×g for 10 min at 4°C. The supernatant was collected, and the protein concentration was determined using the BCA Protein Assay Reagent (Pierce). Total protein (50–80 µg) was subjected to 10–15% sodium dodecyl sulfate-polyacrylamide gel electrophoresis (SDS-PAGE) and was transferred to nitrocellulose membranes (Millipore). After blocking in 5% nonfat dry milk in TBS-T (10 mM Tris-Cl, pH 7.5, 150 mM NaCl, 0.05% Tween 20), the membranes were incubated overnight with primary antibodies at 4°C. The membranes were then washed four times with TBST and then incubated with HRP-conjugated secondary antibodies for 1 h at room temperature. Proteins were visualized using chemiluminescent substrate (Millipore) according to the manufacturer's instructions.

### Ubiquitination assays

For ubiquitination analysis, lentiviral siRNA was transfected into macrophages, and whole-cell extracts were immunoprecipitated with anti-TRAF6 antibody (1 µg). They were then analyzed by immunoblot with anti-Ub, anti-Lys63 specific Ub, and anti-Lys48 specific Ub.

### RT-PCR analysis

Total RNA was extracted with 1 ml of TRIzol reagent (Invitrogen), according to the manufacturer's instructions. Next, 1 µg of total RNA was reverse-transcribed with the ReverTra Ace qPCR RT Kit (FSQ-101; Toyobo), according to the manufacturer's instructions. A LightCycler (LC480; Roche) and a SYBR RT-PCR kit (QPK-212; Toyobo) were used for quantitative real-time RT-PCR analysis. Expression values were normalized to the control gene GAPDH (encoding glyceraldehyde phosphate dehydrogenase). The primers used were: TNF-α: sense 5′-TTCTCATTCCTGCTTGTG-3′ and anti-sense 5′-TTGGTGGTTTGCTACG-3′; IL-6: sense 5′-CTTCTTGGGACTGATG-3′ and anti-sense 5′-CTCATTTCCACGATTT-3′; RANTES: sense 5′-CCATGAAGGTCTCCGCGGC-3′ and anti-sense 5′-TCCTAGCTCATCTCCAAAGA-3; and IP-10: sense 5′-AGTGGCATTCAAGGAGTACC-3′ and anti-sense 5′-ATCCTTGGAAGCACTGCATC-3′.

### Cytokine detection

TNF-α and IL-6 were measured in the supernatant and serum using ELISA kits (MTA00B, M6000B; R&D Systems), according to the manufacturer's instructions. All experiments were performed in duplicate at room temperature. Absorbances were detected at 450 nm using a Varioskan Flash multifunction plate reader (Thermo Scientific).

### Statistical analysis

All data were presented as means ± standard deviation (SD). SPSS for Windows v.16.0 (SPSS Inc., Chicago, IL, USA) was used for the statistical analyses. Statistical significance between two groups was determined using Student's t-test. Data from a single experiment were used to represent three independent experiments with similar results. *P* values <0.05 were considered statistically significant.

## Results

### Knocking down WWP1 enhanced LPS-induced, but not TNF-α –induced, TNF- α and IL-6 production

**Figure 1:**
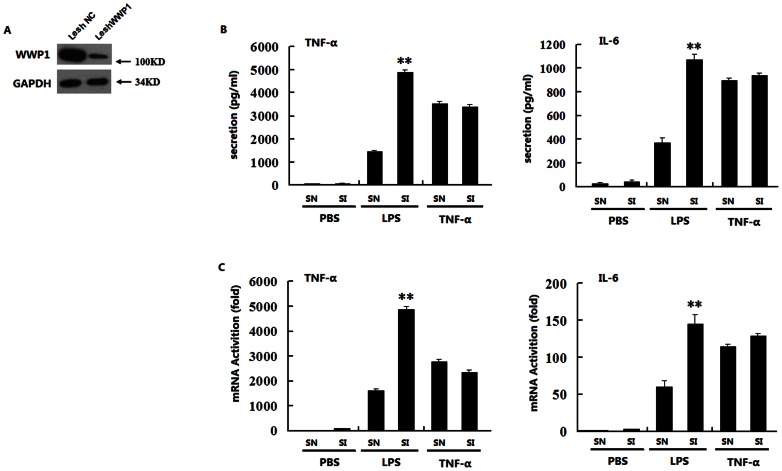
Knocking down WWP1 enhances TNF-α and IL-6 production induced by LPS but not by TNF-α. (A) Effects of WWP1 RNAi (Lesh WWP1) on WWP1 expression. Peritoneal macrophages (2×10^6^) were infected with the Lesh NC and Lesh WWP1 virus particles (m.o.i 40) for 48 h, and immunoblot analysis was then performed with the anti-WWP1 antibody. (B) Effects of WWP1 RNAi (Lesh WWP1) on LPS or TNF-α–induced TNF-α and IL-6 secretion. Peritoneal macrophages (7.5×10^5^) were infected with the Lesh NC and Lesh WWP1 virus particles (m.o.i 40) for 48 h, and then, LPS (at a final concentration of 400 ng/mL) or TNF-α (at a final concentration of 10 ng/mL) was used to stimulate the cells. The medium was changed 60 minutes later to fresh DMEM with 10% FBS, and ELISA analysis was used to detect TNF-α and IL-6 secretion after 12 h of stimulation. (C) Effects of WWP1 RNAi (Lesh WWP1) on LPS or TNF-α induced TNF-α and IL-6 mRNA transcription. Peritoneal macrophages (7.5×10^5^) were infected with the Lesh NC and Lesh WWP1 virus particles (m.o.i 40) for 48 h, and then, LPS (at a final concentration of 400 ng/mL) or TNF-α (at a final concentration of 10 ng/mL) were added to stimulate the cells for 8 h respectively. RT-PCR analysis was used to detect TNF-α and IL-6 mRNA levels. **P<0.01 (t-test), data are representative of three independent experiments (mean and S.D. of three replicates).

To investigate the effect of WWP1 on LPS or TNF-α induced TNF-α and IL-6 production, we constructed a lentivirus containing negative control siRNA (Lesh NC) and a lentivirus containing WWP1 siRNA (Lesh WWP1). These were used to infect cultured peritoneal macrophages. At 48 hours post-infection, immunoblot analyses suggested that Lesh WWP1 could markedly inhibit the expression of endogenous WWP1 (Figure 1A). We next infected peritoneal macrophages with Lesh NC and Lesh WWP1. ELISA analysis indicated that knocking down WWP1 significantly enhanced the LPS-induced TNF-α and IL-6 production, but not the TNF-α-induced production of these proteins (Figure 1B), and RT-PCR analysis confirmed these results (Figure 1C). Collectively, this data suggests that knocking down WWP1 enhanced TNF-α and IL-6 production induced by LPS, but not TNF-α.

### Over-expression of WWP1 inhibits LPS-induced, but not TNF-α-induced, TNF-α and IL-6 production

**Figure 2:**
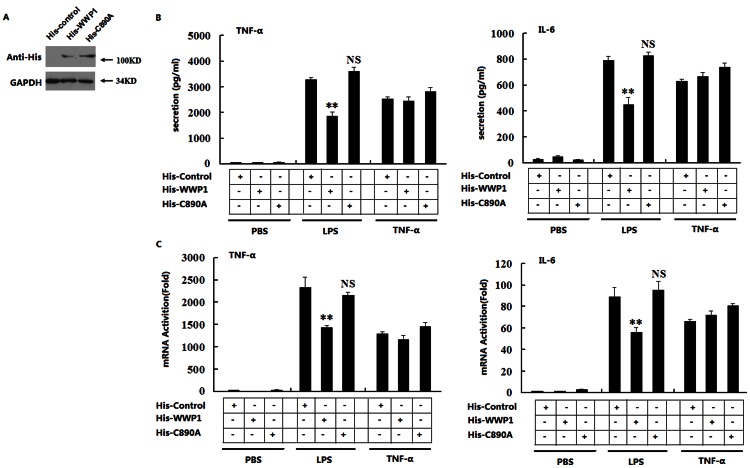
Over-expression of WWP1 inhibits TNF-α and IL-6 production induced by LPS, but not by TNF-α, through its C-terminal catalytic activity. (A) Over-expression of WWP1 and C890A in RAW164.7 stable cell lines. Western blots were used to analyze the His-WWP1 and His-C890A expression in RAW264.7 cells stably transfected with expression plasmid using the His antibody. (B) Effects of over-expression of wild type WWP1 and C890A on LPS- or TNF-α-induced TNF-α and IL-6 secretion. The RAW264.7 stable cell lines of WWP1 and C890A (4×10^5^) were stimulated by LPS (at a final concentration of 400 ng/mL) or TNF-α (at a final concentration of 10 ng/mL) for 12 h. ELISA analysis was used to detect the levels of TNF-α and IL-6 secretion. (C) Effects of over-expression of wild type WWP1 and C890A on LPS- or TNF-α-induced TNF-α and IL-6 transcription. The RAW264.7 stable cell lines of WWP1 and C890A (4×10^5^) were stimulated by LPS (at a final concentration of 400 ng/mL) or TNF-α (at a final concentration of 10 ng/mL) for 8 h. RT-PCR analysis was used to detect TNF-α and IL-6 transcription. **P<0.01 (t-test). Data are representative of three independent experiments (mean and S.D. of three replicates).

WWP1 has been reported as an important E3 ubiquitin ligase in a variety of cellular biological processes, and its C-terminal catalytic activity is essential for the transfer of ubiquitin. We investigated whether the over-expression of WWP1 would change the TNF-α and IL-6 production induced by LPS or TNF-α through E3 ubiquitin ligase activity. We constructed pcDNA3.1-His plasmids that expressed wild type WWP1 protein (His-WWP1) or WWP1-C890A mutant protein (His-C890A). The mutant WWP1-C890A protein has no catalytic activation.^23^ Immunoblot analysis of RAW264.7 stable cell lines indicated that these cells expressed His-Control, His-WWP1, and His-C890A very well (Figure 2A). These cells were stimulated with LPS or TNF-α for 12 h, after which ELISA analysis was performed. We found that over-expression of wide type WWP1 (His-WWP1) inhibited the LPS- but not TNF-α-induced TNF-α and IL-6 production. The WWP1-C890A mutant protein (His-C890A), however, did not have the same effects (Figure 2B). RT-PCR analysis confirmed these results (Figure 2C).

RT-PCR analysis of co-transfections of TRIF or MyD88 with His-Control, His-WWP1, and His-C890A showed that WWP1 could influence MyD88- but not TRIF-mediated IFN-β production (S1A). We next infected peritoneal macrophages with Lesh NC and Lesh WWP1. RT-PCR analysis indicated that knocking down WWP1 did not influence LPS-induced production of RANTES and IP 10, two IFN-inducible genes (S1B). This data suggested that over-expression of WWP1 inhibited the TNF-α and IL-6 production induced by LPS, but not by TNF-α, and that its E3 ubiquitin ligase activity played an important role in this process.

### WWP1 inhibits IκB-α, NF-κB, and MAPK activation stimulated by LPS

**Figure 3:**
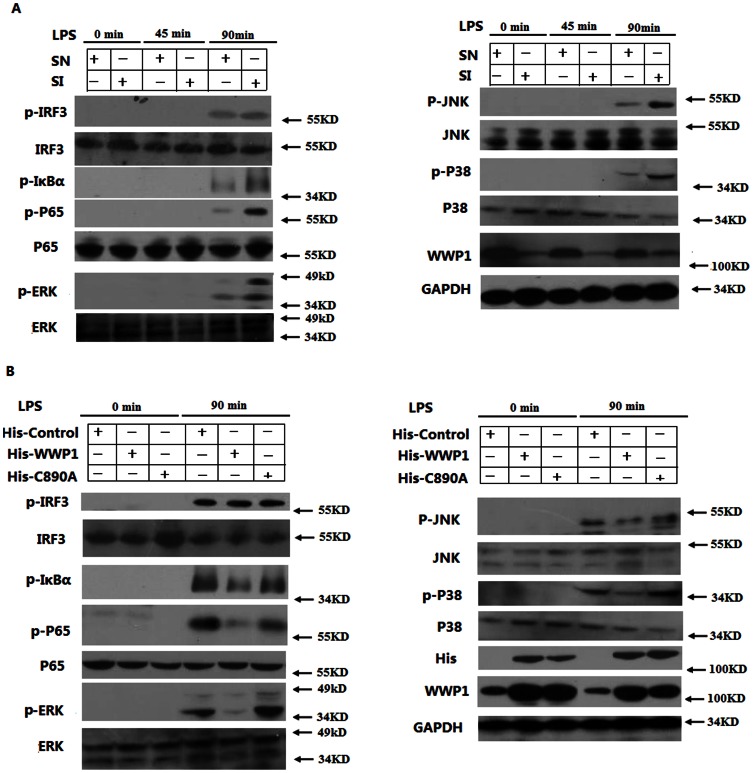
WWP1 inhibits IκB-α, NF-κB, and MAPK, but not IRF3, activation by LPS stimulation. (A) Knocking down WWP1 enhanced LPS-induced phosphorylation of IκB-α, NF-κB and MAPK, but not of IRF3. Peritoneal macrophages (3×10^6^) were infected with the Lesh NC and Lesh WWP1 virus particles (m.o.i 40) for 48 h, and then, LPS was used (at a final concentration of 400 ng/mL) to stimulate the cells for the indicated times. The lysates were then subsequently analyzed by immunoblots with the indicated antibodies. (B) Over-expression of WWP1 inhibited LPS–induced phosphorylation of IκB-α, NF-κB and MAPK. The RAW264.7 stable cell lines of WWP1 and C890A (4×10^5^) were stimulated by LPS (at a final concentration of 400 ng/mL) for the indicated times. The lysates were then subsequently analyzed by immunoblots with the indicated antibodies. Data are from one experiment representative of three independent experiments with similar results.

LPS is known to stimulate the TLR4-mediated production of TNF-α and IL-6 primarily through the MyD88-dependent pathway. In this pathway, the activation and phosphorylation of IκB-α, NF-κB, and MAPK (through JNK, ERK, and P38) are essential for the transcription of TNF-α and IL-6 mRNA. The phosphorylation of IRF3 also plays an important role in the production of TNF-α and IL-6 through the TRIF-dependent pathway. In order to investigate whether WWP1 inhibited the phosphorylation of IRF3, IκB-α, NF-κB and MAPK through the TLR4 pathways, we infected cultured peritoneal macrophages with Lesh NC, Lesh WWP1 and then stimulated the cells with LPS. Western blotting showed that knocking down WWP1 increased the LPS-induced phosphorylation of IκB-α, NF-κB and MAPK, including increased p-JNK, p-ERK, p-P38, particularly at the 90-minute time point post-treatment. Interestingly, however, knocking down WWP1 did not affect the phosphorylation of IRF3 (Figure 3A).

To further confirm the role of WWP1 and its E3 ubiquitin ligase activity in TLR signaling, we treated RAW264.7 cell lines, which continuously overexpressed His-Control, His-WWP1, and His-C890A, with LPS. Western blotting showed that full-length WWP1, but not WWP1-C890A mutant protein, significantly inhibited the LPS-induced activation of IκB-α, NF-κB, and MAPK by reducing the phosphorylation of JNK, ERK, and P38, but not IRF3 (Figure 3B). These data suggest that WWP1 inhibits LPS-induced activation and phosphorylation of IκB-α, NF-κB and MAPK, but not IRF3, and that its E3 ubiquitin ligase activity is necessary for these activities.

### WWP1 interacts with TRAF6, but not IRAK1, *in vitro* and *in vivo*

**Figure 4:**
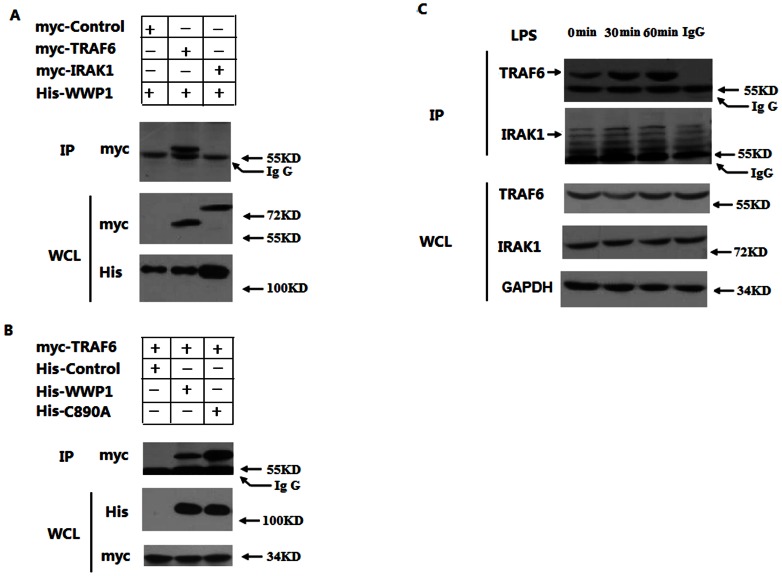
WWP1 interacts with TRAF6, but not IRAK1, both *in vitro* and *in vivo*. (A) WWP1 interacted with TRAF6, but not IRAK1, in the mammalian over-expression system. HEK293T cells (2×10^6^) were transfected with the His-WWP1 (3 µg) plasmids and Myc-TRAF6 or Myc-IRAK1 plasmids (3 µg). After 24 h, whole cell extracts were immunoprecipitated with anti-His and then analyzed with anti-Myc. Cell lysates were analyzed by immunoblotting with anti-Myc and anti-His. (B) Both wild type WWP1 and C890A could interact with TRAF6 in the mammalian over-expression system. HEK293T cells (2×10^6^) were transfected with the myc-TRAF6 plasmids and His-WWP1 (3 µg) or His- C890A plasmids (3 µg). After 24 h, whole cell extracts were immunoprecipitated with anti-His, and then analyzed with anti-Myc. Cell lysates were analyzed by immunoblotting with anti-Myc and anti-His. (C) Endogenous WWP1 was associated with TRAF6 following LPS stimulation. RAW264.7 cells (3×10^7^) were left untreated or stimulated with LPS (at a final concentration of 400 ng/mL) for the indicated times. Whole cell extracts were immunoprecipitated with IgG and anti-WWP1, and then analyzed with anti-TRAF6 or anti-IRAK1. Cell lysates were also analyzed by immunoblotting with anti-TRAF6 or anti-IRAK1. Data are from one experiment representative of three independent experiments with similar results.

To investigate the regulatory mechanisms of WWP1 in LPS–induced signaling pathways, we examined the interactions of WWP1 with different signaling components of these pathways. Our previous results suggested that WWP1 might interact with upstream molecules in MyD88 signaling, such as TRAF6, but not with TLR4 (S2) or IRAKs. We co-expressed His-WWP1 with Myc-TRAF6 and Myc-IRAK1 in HEK293T cells. Immunoprecipitation and immunoblot analyses revealed that His-WWP1 associated with Myc-TRAF6 but not with Myc-IRAK1 (Figure 4A, S3). Next, we investigated the role of the catalytic activity of WWP1 in its interaction with TRAF6. We co-expressed Myc-TRAF6 with His-WWP1 and His-C890A in HEK293T cells. Immunoprecipitation and immunoblot analyses indicated that both His-WWP1 and His-C890A were able to associate with Myc-TRAF6 (Figure 4B). Thus, both the wild type WWP1 protein and WWP1-C890A mutant protein could interact with TRAF6 normally *in vitro*. Therefore, the catalytic activity of WWP1 is not essential for these interactions.

We further investigated this interaction through endogenous co-immunoprecipitation experiments. WWP1 was weakly associated with TRAF6 in untreated RAW264.7 cells, and this level of association was significantly higher at 30 minute and 60 minute time points post-LPS treatment (Figure 4C), and with the time going on, this interaction ability was weaken especially in 12 h (S4).These results suggest that LPS promotes the endogenous association of WWP1 with TRAF6, but not IRAK1.

### WWP1 degrades TRAF6, but not IRAK1, by the proteasome pathway

**Figure 5:**
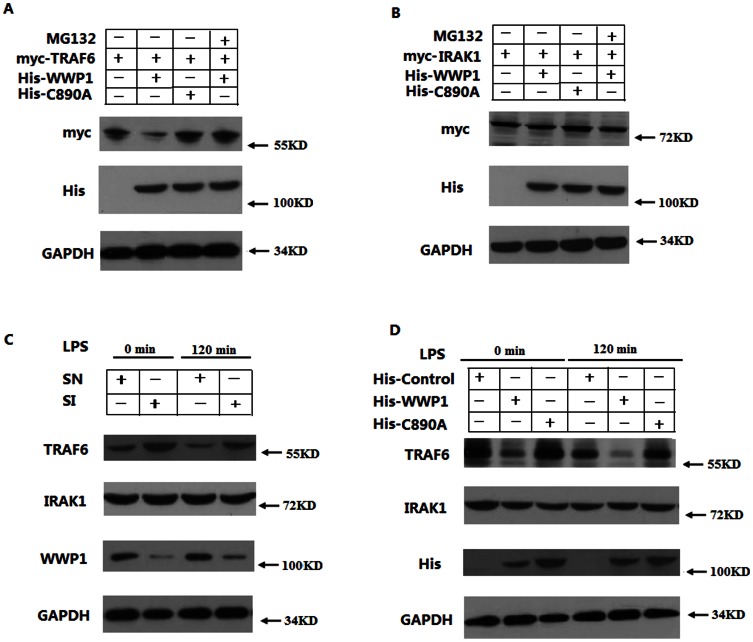
WWP1 degrades TRAF6, but not IRAK1, by the proteasome pathway. (A, B) WWP1 could degrade TRAF6, but not IRAK1, *in vitro*. HEK293T cells were co-transfected with Myc-TRAF6 (3 µg) (A) or IRAK1 (3 µg) (B) and His-WWP1(3 µg) or His-C890A plasmids (3 µg). They were then treated with MG132. Western blots were used to analyze the protein levels with anti-Myc. (C) Knocking down WWP1 enhanced the expression of TRAF6 *in vivo*. Peritoneal macrophages were infected with Lesh NC and Lesh WWP1 virus particles (m.o.i 40) for 48 h, and then stimulated with LPS (at a final concentration of 400 ng/mL). Cell lysates were also analyzed by immunoblotting with anti-TRAF6 or anti-IRAK1. (D) Over-expression of WWP1 inhibited the expression of TRAF6 *in vivo*. The RAW264.7 stable cell lines of WWP1 and C890A (4×10^5^) were stimulated by LPS (at a final concentration of 400 ng/mL) for the indicated times. The lysates were then subsequently analyzed by immunoblots with the indicated antibodies. Data are from one experiment representative of three independent experiments with similar results.

As an important E3 ubiquitin ligase, it is possible that WWP1 could ubiquitinate and target proteins for degradation by the proteasome pathway. To investigate whether WWP1 signaling results in TRAF6 degradation, we co-transfected His-WWP1 and His-C890A with Myc-TRAF6 in HEK293T cells. Immunoblot analyses showed that wild type WWP1 promotes the degradation of TRAF6. Additionally, this degradation was abolished when we added the proteasome inhibitor MG132 to the media. Furthermore, the WWP1-C890A mutant protein (His-C890A) did not promote degradation of TRAF6 (Figure 5A). We also co-transfected His-WWP1 and His-C890A with Myc-IRAK1 in HEK293T cells, but the expression level of the Myc-IRAK1 did not change (Figure 5B). Collectively, this data suggests that WWP1 can degrade TRAF6, but not IRAK1, by the proteasome pathway *in vitro*.

To further investigate the effect of WWP1 on TRAF6 *in vivo*, we infected cultured peritoneal macrophages with Lesh NC and Lesh WWP1. Immunoblot experiments show that knocking down WWP1 resulted in an increase in endogenous TRAF6, but not IRAK1, expression and that, when the infected peritoneal macrophages were treated with LPS, TRAF6 expression was even more highly expressed in Lesh WWP1 macrophages (Figure 5C). Endogenous TRAF6 and IRAK1 expression levels were also analyzed in RAW264.7 stable cell lines in which His-Control, His-WWP1, and and His- C890A were continuously overexpressed. We found that endogenous TRAF6, but not IRAK1, levels were much lower in His-WWP1 cell lines in comparison to the His-Control and His-C890A cell lines (Figure 5D). This data indicates that WWP1 degrades TRAF6 but not IRAK1 by the proteasome pathway *in vivo*.

### WWP1 influences LPS-induced K48-linked, but not K63-linked, polyubiquitination of endogenous TRAF6

**Figure 6:**
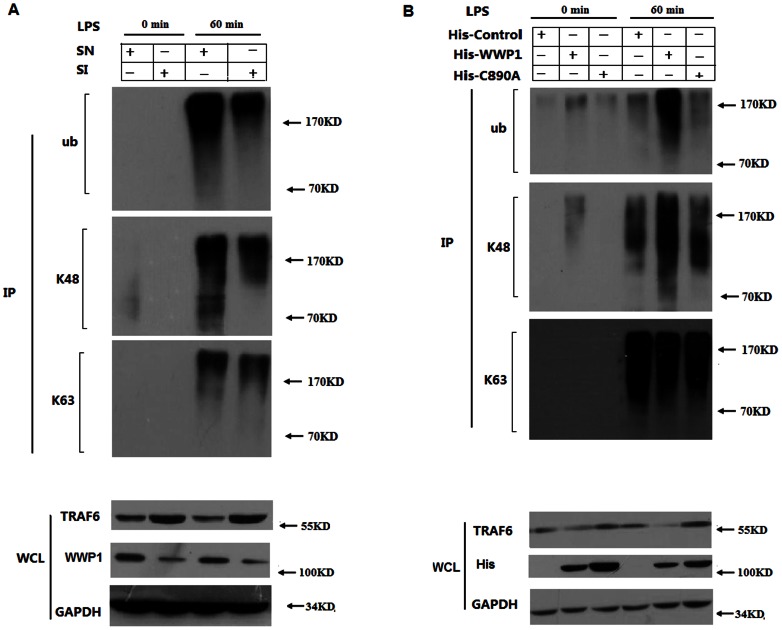
Knocking down WWP1 reduces LPS–induced K48-linked, but not K63-linked, polyubiquitination of endogenous TRAF6. (A) Peritoneal macrophages (2×10^6^) were infected with the Lesh NC and Lesh WWP1 virus particles (m.o.i 40) for 48 h, and then treated with LPS (at a final concentration of 400 ng/mL) for the indicated times. Whole cell lysates were subjected to immunoprecipitation with anti-TRAF6, followed by Western blot analysis with anti-Ub, anti-Lys48 specific Ub and anti-Lys63 specific Ub. Cell lysates were also analyzed by immunoblotting with anti-TRAF6 or anti-WWP1. Data are from one experiment representative of three independent experiments with similar results. (B) The RAW264.7 stable cell lines of WWP1 and C890A (4×10^5^) were stimulated by LPS (at a final concentration of 400 ng/mL) for the indicated times. Whole cell lysates were subjected to immunoprecipitation with anti-TRAF6 followed by Western blot analysis with anti-Ub, anti-Lys48 specific Ub and anti-Lys63 specific Ub. Cell lysates were also analyzed by immunoblotting with anti-TRAF6 or anti-WWP1. Data are from one experiment representative of three independent experiments with similar results.

To determine whether endogenous WWP1 is required for LPS-induced TRAF6 polyubiquitination, we infected peritoneal macrophages with Lesh NC and Lesh WWP1. Immunoprecipitation experiments indicated that TRAF6 was polyubiquitinated by both K48- and K63-linked mechanisms when cells were stimulated by LPS. Furthermore, knocking down WWP1 reduced LPS-induced TRAF6 polyubiquitination. Next, we used the antibodies specific to Lys48 Ub and Lys63 Ub to determine whether endogenous WWP1 mediates K48- or K63- linked polyubiquitination. These experiments showed that knocking down WWP1 reduced LPS-induced K48-linked, but not K63-linked, polyubiquitination of TRAF6 (Figure 6A). These results suggest that WWP1 mediates the LPS-induced K48-linked polyubiquitination of endogenous TRAF6.

We further investigated the role of WWP1 on LPS-induced polyubiquitination of TRAF-6 and whether this activity is due to WWP1 functioning as an E3 ubiquitin ligase. We treated RAW264.7 cell lines, which continuously overexpressed His-Control, His-WWP1 and His-C890A, with LPS. Immunoprecipitation experiments showed that TRAF6 was polyubiquitinated in both a K48- and K63-linked manner when cells were treated with LPS. Additionally, the over-expression of WWP1 enhanced the LPS-induced TRAF6 polyubiquitination. Next, we used specific antibodies specific to Lys48 Ub and Lys63 Ub to determine whether endogenous WWP1 mediates K48- or K63- linked polyubiquitination. Our results indicate that over-expression of WWP1, but not the C890A mutant, enhanced K48-linked, but not K63-linked, LPS-induced TRAF6 polyubiquitination of TRAF6 (Figure 6B). These results suggest that WWP1 mediates LPS-induced K48-linked polyubiquitination of endogenous TRAF6 through its E3 ligase activity.

## Discussion

Toll-like receptors (TLRs) play important roles in both innate and adaptive immunity. TLRs recognize specific pathogen-associated molecular patterns (PAMPs) on microbes and activate signaling pathways that provide specific immunological responses tailored for the microbes expressing these molecules. The specific responses initiated by individual TLRs depends on the recruitment of a single or specific combination of TIR-domain-containing adaptor protein(s) (e.g., MyD88, TRIAP, TRIF, or TRAM) [1], [2].

TLR4 is the only TLR that recruits four adaptor proteins and activates two distinct signaling pathways: the MyD88-dependent and TRIF-dependent pathways [2], [27]. These pathways have different kinetics. TLR4 first recruits TRIAP and MyD88 to the site of infection. TRIAP localizes to the plasma membrane via its interaction with PIP_2_ and then serves to bridge interactions between MyD88 and TLR4 upon LPS engagement [28]. MyD88 recruits IRAKs, TRAF6, and the TAK1 complex, leading to early-phase activation of NF-κB and MAP kinases [1], [2], [27]. TLR4 is then endocytosed and delivered to intracellular vesicles to form a complex with TRAM and TRIF which recruits TRAF3 and the protein kinases TBK1 and IKKi. These kinases catalyze the phosphorylation of IRF3, leading to the expression of type I IFN [11], [12]. In this study, we demonstrate that WWP1 is a negative regulator of TLR4-mediated production of TNF-α and IL-6. Our results show that WWP1 can bind to TRAF6 but not IRAK1, even following LPS stimulation. TLRs often share common adaptors, thus, the potential involvement of WWP1 in other TLR (TLR2, TLR6) activated signaling pathways requires further investigation.

Although the activation of TLR signaling and the resulting secretion of proinflammatory cytokines is important for the elimination of invading microorganisms, uncontrolled activation of these pathways may lead to autoimmune and inflammatory diseases [4]. Therefore, TLR signaling must be tightly controlled to prevent excessive inflammatory responses. Recent studies have highlighted the importance of ubiquitination in modulating the innate immune response to invading pathogens via the TLR pathways [29]. A20 is a protein induced during TLR stimulation that has two enzymatic activities where it can act as both an E3 ubiquitin ligase and a deubiquitinase. *In vitro* analyses have shown that A20 restricts NF-κB activation by modulating RIP1 and TRAF6 [30]. Shi et al. (2007) identified TRIM30α as, whose expression was upregulated by various TLR agonists, including LPS, as a negative regulator of TLR4 signaling [31]. They reported that TRIM30α interacted with TAK1, TAB2 and TAB3 and induced the degradation of TAB2 and TAB3.

WWP1 interacts with a number of substrate proteins by inducing their ubiquitination and lysosomal degradation and, consequently, regulates a variety of cellular biological processes. Lee et al. (2013) reported that WWP1 interacted with AMPK and downregulated its expression through ubiquitin ligase activity in skeletal muscle [32]. Subik et al. (2012) demonstrated that WWP1 functioned as an inhibitor of breast cancer metastasis to bone by negatively regulating CXCL 12-induced lysosomal degradation of CXCR4 [33]. In this study, we show for the first time that WWP1 plays an important role in TLR-mediated inflammation as an E3 ubiquitin ligase. Knocking down WWP1 expression by siRNA resulted in augmented activation of NF-κB and MAPKs and enhanced expression of proinflammatory cytokines such as TNF-α and IL-6. Over-expression of WWP1 in RAW264.7 cells had the opposite effects. When we stimulated cells with LPS, WWP1 functioned as an E3 ligase by binding to TRAF6 and promoting K48-linked polyubiquitination, which led to the proteasomal degradation of TRAF6. Consistently, knocking down WWP1 expression resulted in lower K48-linked polyubiquitination and higher protein levels of TRAF6 in peritoneal macrophages. We showed that over-expression of WWP1 in RAW264.7 stable cell lines resulted in lower protein levels of TRAF6, while the over-expression of the catalytically inactive mutant WWP1-C890A (His-C890A) did not change the protein levels of TRAF6. We have previously established that activation of TLR does not upregulate the expression of WWP1 (S5). Thus, the modification and translocation of WWP1 is most likely regulated by LPS stimulation.

In conclusion, when we treated cells with LPS, the E3 ligase WWP1 bound to TRAF6 and promoted its K48-linked polyubiquitination, leading to the proteasomal degradation of TRAF6. These findings demonstrate that WWP1 plays an important role as a negative regulator in TLR4-mediated production of TNF-α and IL-6.

## Supporting Information

Figure S1 **WWP1 does not influence TRIF-mediated IFN-beta production.** (A) HEK293T cells (2×10^6^) were transfected with the His-Control or His-WWP1 (3 µg) plasmids and TRIF-plasmid or MyD88 plasmids (3 µg). After 24 h, RT-PCR analysis was used to detect IFN-beta mRNA levels. **P<0.01 (t-test). Data are representative of three independent experiments (mean and S.D. of three replicates). (B) Peritoneal macrophages (7.5×10^5^) were infected with the Lesh NC and Lesh WWP1 virus particles (m.o.i 40) for 48 h, and then LPS (at a final concentration of 400 ng/mL) were for 8 h added to stimulate the cells. RT-PCR analysis was used to detect the RANTES and IP 10 mRNA levels. **P<0.01 (t-test). Data are representative of three independent experiments (mean and S.D. of three replicates).
(TIF)

Figure S2 **WWP1 does not interact with or degrade TLR4.** (A) HEK293T cells (2×10^6^) were transfected with the His-WWP1 (3 µg) plasmids and Myc-TLR4 plasmids (3 µg). After 24 h, whole cell extracts were immunoprecipitated with anti-HA and then analyzed with anti-His and anti-HA. Cell lysates were analyzed by immunoblotting with anti-HA and anti-His. (B) Peritoneal macrophages (2×10^6^) were infected with the Lesh NC and Lesh WWP1 virus particles (m.o.i 40) for 48 h, and then treated with LPS (at a final concentration of 400 ng/mL) for the indicated times. Whole cell lysates were subjected to immunoprecipitation with anti-WWP1 followed by Western blot analysis with anti-TLR4 and anti-WWP1. Cell lysates were also analyzed by immunoblotting with anti-TLR4 or anti-actin. Data are from one experiment representative of three independent experiments with similar results.
(TIF)

Figure S3 **WWP1 interacts with TRAF6, but not IRAK1, in the mammalian over-expression system.** HEK293T cells (2×10^6^) were transfected with the His-WWP1 (3 µg) plasmids and Myc-TRAF6 or Myc-IRAK1 plasmids (3 µg). After 24 h, whole cell extracts were immunoprecipitated with anti-Myc and then analyzed with anti-His. Cell lysates were analyzed by immunoblotting with anti-Myc and anti-His.
(TIF)

Figure S4 **WWP1 interactes with TRAF6 but not IRAK1 both** ***in vivo*** **.** RAW 264.7 cells (3×10^7^) were left untreated or stimulated with LPS (at a final concentration of 400 ng/ml) for the indicated times. Whole cell extracts were immunoprecipitated with IgG and anti-WWP1 Ab, and then analyzed with anti-TRAF6 Ab or anti-IRAK1 Ab. Cell lysates were also analyzed by immunoblotting with anti-TRAF6 Ab or anti-IRAK1 Ab. Data are from one experiment representative of three independent experiments with similar results.
(TIF)

Figure S5 **WWP1 production is not influenced by LPS stimulation.** The RAW264.7 cells (4×10^5^) were stimulated by LPS (at a final concentration of 400 ng/mL) for 0, 0.5, 4, 12, and 24 h. Western blotting and RT-PCR analysis were used to detect WWP1 expression. **P<0.01 (t-test). Data are representative of three independent experiments (mean and S.D. of three replicates).
(TIF)
