# Supplementary figures and images for: WW Domain Containing E3 Ubiquitin Protein Ligase 1 (WWP1) Negatively Regulates TLR4-Mediated TNF-α and IL-6 Production by Proteasomal Degradation of TNF Receptor Associated Factor 6 (TRAF6)
Source: PLoS One. 2013 Jun 17;8(6):e67633. doi: 10.1371/journal.pone.0067633 (PMC3684580; doi:10.1371/journal.pone.0067633)

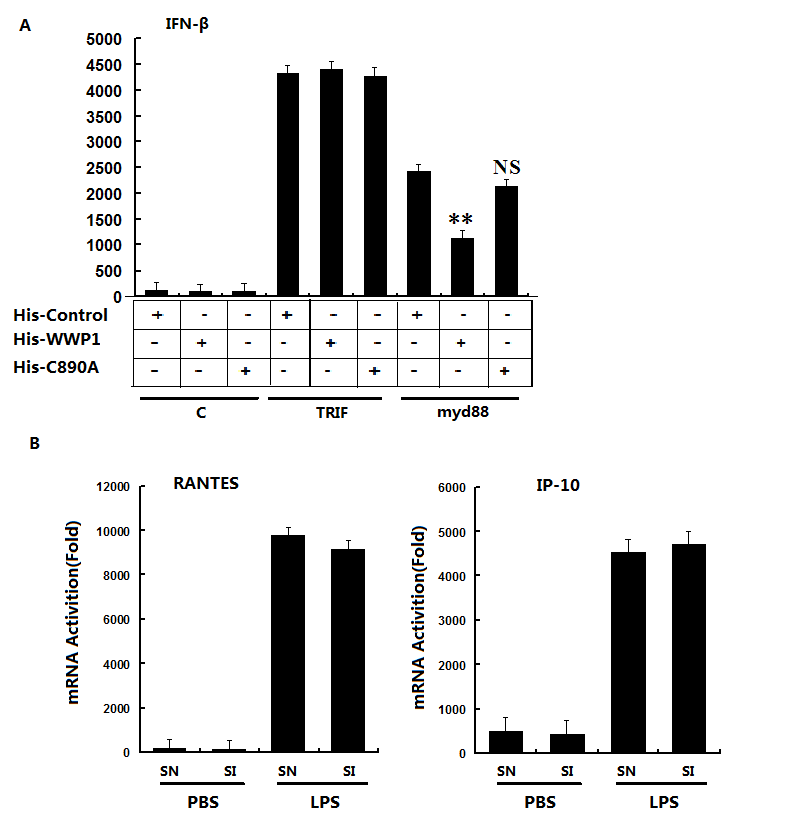

Supplement: Figure S1 — WWP1 does not influence TRIF-mediated IFN-beta production. (A) HEK293T cells (2×10^6^) were transfected with the His-Control or His-WWP1 (3 µg) plasmids and TRIF-plasmid or MyD88 plasmids (3 µg). After 24 h, RT-PCR analysis was used to detect IFN-beta mRNA levels. **P<0.01 (t-test). Data are representative of three independent experiments (mean and S.D. of three replicates). (B) Peritoneal macrophages (7.5×10^5^) were infected with the Lesh NC and Lesh WWP1 virus particles (m.o.i 40) for 48 h, and then LPS (at a final concentration of 400 ng/mL) were for 8 h added to stimulate the cells. RT-PCR analysis was used to detect the RANTES and IP 10 mRNA levels. **P<0.01 (t-test). Data are representative of three independent experiments (mean and S.D. of three replicates). (TIF) [file pone.0067633.s001.tif]

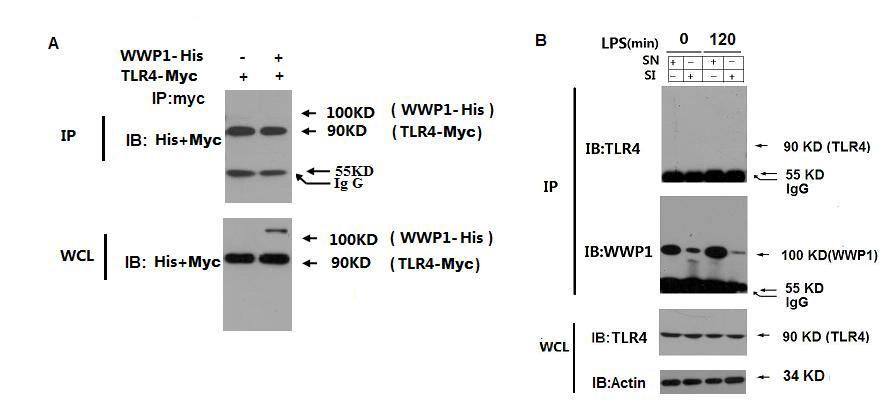

Supplement: Figure S2 — WWP1 does not interact with or degrade TLR4. (A) HEK293T cells (2×10^6^) were transfected with the His-WWP1 (3 µg) plasmids and Myc-TLR4 plasmids (3 µg). After 24 h, whole cell extracts were immunoprecipitated with anti-HA and then analyzed with anti-His and anti-HA. Cell lysates were analyzed by immunoblotting with anti-HA and anti-His. (B) Peritoneal macrophages (2×10^6^) were infected with the Lesh NC and Lesh WWP1 virus particles (m.o.i 40) for 48 h, and then treated with LPS (at a final concentration of 400 ng/mL) for the indicated times. Whole cell lysates were subjected to immunoprecipitation with anti-WWP1 followed by Western blot analysis with anti-TLR4 and anti-WWP1. Cell lysates were also analyzed by immunoblotting with anti-TLR4 or anti-actin. Data are from one experiment representative of three independent experiments with similar results. (TIF) [file pone.0067633.s002.tif]

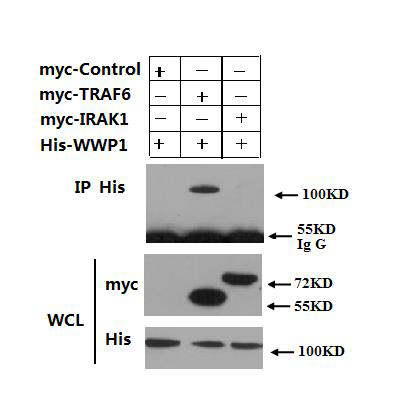

Supplement: Figure S3 — WWP1 interacts with TRAF6, but not IRAK1, in the mammalian over-expression system. HEK293T cells (2×10^6^) were transfected with the His-WWP1 (3 µg) plasmids and Myc-TRAF6 or Myc-IRAK1 plasmids (3 µg). After 24 h, whole cell extracts were immunoprecipitated with anti-Myc and then analyzed with anti-His. Cell lysates were analyzed by immunoblotting with anti-Myc and anti-His. (TIF) [file pone.0067633.s003.tif]

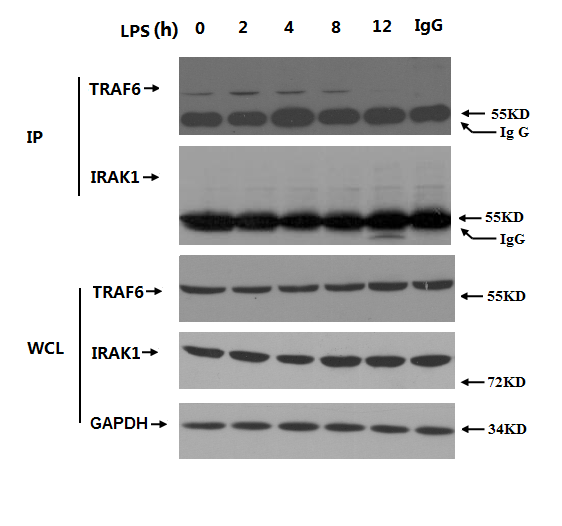

Supplement: Figure S4 — WWP1 interactes with TRAF6 but not IRAK1 both in vivo . RAW 264.7 cells (3×10^7^) were left untreated or stimulated with LPS (at a final concentration of 400 ng/ml) for the indicated times. Whole cell extracts were immunoprecipitated with IgG and anti-WWP1 Ab, and then analyzed with anti-TRAF6 Ab or anti-IRAK1 Ab. Cell lysates were also analyzed by immunoblotting with anti-TRAF6 Ab or anti-IRAK1 Ab. Data are from one experiment representative of three independent experiments with similar results. (TIF) [file pone.0067633.s004.tif]

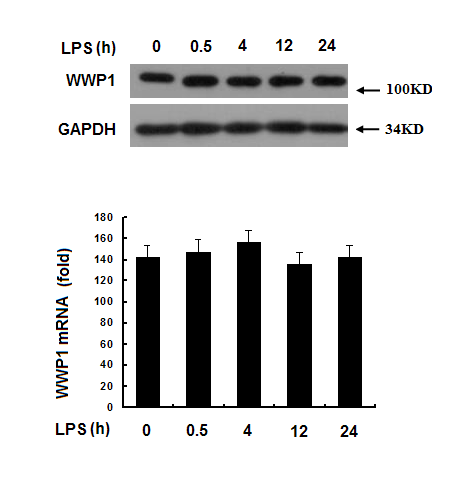

Supplement: Figure S5 — WWP1 production is not influenced by LPS stimulation. The RAW264.7 cells (4×10^5^) were stimulated by LPS (at a final concentration of 400 ng/mL) for 0, 0.5, 4, 12, and 24 h. Western blotting and RT-PCR analysis were used to detect WWP1 expression. **P<0.01 (t-test). Data are representative of three independent experiments (mean and S.D. of three replicates). (TIF) [file pone.0067633.s005.tif]
